# Prevention and treatment of malaria in pregnancy: what do pregnant women and health care workers in East India know and do about it?

**DOI:** 10.1186/s12936-018-2339-9

**Published:** 2018-05-18

**Authors:** Lora Sabin, Evan M. S. Hecht, Mohamad I. Brooks, Mrigendra P. Singh, Kojo Yeboah-Antwi, Abanish Rizal, Blair J. Wylie, Philip A. Bondzie, Matthew Banos, Jordan Tuchman, Neeru Singh, Davidson H. Hamer

**Affiliations:** 1 Department of Global Health, Boston University School of Public Health, 801 Massachusetts Ave, Boston, MA USA; 2 Pathfinder, International, 9 Galen Street, Suite 217, Watertown, MA USA; 3 ICMR-National Institute of Malaria Research Field Station, IDVC Mission Medical College Campus, Jabalpur, India; 4 Harvard University, Massachusetts Hall, Cambridge, MA USA; 5 Department of Obstetrics, Gynecology, and Reproductive Biology, Beth Israel Deaconess Medical Center, 330 Brookline Ave, Boston, MA USA; 6 Boston University School of Medicine, 72 East Concord St, Boston, MA USA; 7 Abt Associates, 4550 Montgomery Ave, #800N, Bethesda, MA USA; 8 Section of Infectious Diseases, Department of Medicine, Boston University School of Medicine, Boston, MA USA

**Keywords:** Malaria in pregnancy, Malaria prevention, Malaria treatment, Traditional remedies, Qualitative research, India, Neem

## Abstract

**Background:**

Limited qualitative research has been performed in India to investigate views and behaviours of pregnant women regarding malaria despite the threat of malaria-related adverse maternal and neonatal outcomes. To address this gap, a comprehensive study on malaria prevention and treatment attitudes, knowledge and behaviour among pregnant women in India was conducted.

**Methods:**

Pregnant women and healthcare workers (HCWs), encompassing clinic-based providers, traditional birth attendants, and auxiliary nurse-midwives were enrolled for in-depth interviews (IDIs) at 7 hospital sites and nearby communities in Jharkhand and Chhattisgarh States. Questions addressed health concerns and attitudes, knowledge and practices regarding malaria prevention and treatment; probing covered modern and traditional approaches. Data were analyzed using a thematic approach.

**Results:**

A total of 83 pregnant women and 119 HCWs participated in 202 IDIs, 90 in Jharkhand and 112 in Chhattisgarh. A majority of Jharkhand respondents, but only one-fourth in Chhattisgarh, named malaria among top health issues for pregnant women. Just over half of pregnant women were willing to try new prevention methods (especially insecticide-treated bed nets), although cost-related barriers to such methods were stressed. Most respondents voiced concerns about malaria treatment during pregnancy, mainly citing potential harm to the baby. Most knew that mosquitoes transmitted malaria, but a substantial minority, including among HCWs, described incorrect transmission modes. Most knew a proven prevention method (usually bed nets or coils); a few knew other methods. A minority of pregnant women, but most HCWs, knew about malaria treatment, although some HCWs described unproven treatments. Most respondents described use of modern prevention methods in their communities, typically bed nets, although probing revealed irregular use. Half (especially in Jharkhand and particularly HCWs) described use of traditional prevention approaches such as burning leaves and rubbing oils on the body; traditional remedies for malaria treatment were common, and varied by site and population.

**Conclusions:**

Understanding of malaria varied as a concern for pregnant women, continued use of unproven malaria prevention and treatment strategies was evident in this population in India. These results highlight the need to educate both pregnant women and HCWs about effective malaria methods to protect pregnant women and their babies from malaria.

## Background

Data from the World Malaria Report 2017 provide reasons for optimism as well as concern. Due to enormous investments in malaria prevention, the report highlights a substantial decline in incidence globally between 2000 and 2016 [1]. Yet, malaria continues to be the leading parasitic cause of death worldwide, with 445,000 deaths from malaria worldwide in 2016 [1]. In some parts of the world, malaria incidence is also increasing; notably, malaria infections have increased in number in the two most recent years of data (2015 and 2016) [1]. In South Asia, India is home to two-thirds of all malaria episodes and deaths [2]. India is also the only country outside of sub-Saharan Africa to be included among the 15 countries where 80% of malaria deaths occurred globally [1]. Although estimated malaria cases declined from 21.1 million to 11.5 million between 2010 and 2013, infections rose to 13.2 million in 2016 [1], and 95% of India’s population remains at risk of infection [3]. Malaria is endemic in India, with most cases reported in eastern and central parts of the country [4], and is characterized by significant complexities due to multicultural settings, diverse healthcare systems and geo-ecological states, seasonality and multidrug-resistant parasites [5, 6]. Malaria poses an enormous financial burden of roughly US$2.0 billion annually in India due to lost earnings [7]. To date, investment in malaria prevention and treatment in India has been relatively modest [1, 8], with some experts fearing that India’s slow pace in preventing malaria may pose a challenge to global malaria elimination [4].

Malaria poses a particular threat to pregnant women, for whom adverse associations have been found between malaria and maternal and neonatal outcomes, including maternal anaemia, stillbirths, preterm labour, and low birth weight [9–14]. The World Health Organization (WHO) has recommended measures to curb morbidity and mortality among pregnant women, focusing on use of insecticide-treated bed nets (ITNs), intermittent preventive treatment (IPT) with sulfadoxine-pyrimethamine, and prompt diagnosis and treatment of infection [14, 15]. A recent review of the burden, pathology, and costs of malaria in pregnancy documents how such measures have contributed to prevention efforts, while highlighting the continued high cost of malaria in pregnancy to national budgets and pregnant women themselves [16].

India’s guidelines for the diagnosis and treatment of malaria in 2013 and 2014 repeated previous encouragement for “use of personal protection measures like insecticide-treated bed nets” for pregnant women and other vulnerable populations [17–19]. Although a 5-year strategic plan for malaria control launched in 2012 noted that provision of ITNs and long-lasting insecticide-treated bed nets (LLINs) to pregnant women and integration of malaria-prevention with antenatal care (ANC) services was being implemented in some districts [20], no specific recommendations for pregnant women were provided in India’s 2016 national plan for malaria elimination by 2030 [21].

In 2007-09, to better understand the views and behaviours of pregnant women in India regarding malaria, a qualitative study of the attitudes, knowledge and practices in this population was conducted in Jharkhand, a state in eastern India [22]. The study was a major component of a larger research project on the burden of malaria in pregnant women [13]. The study found that most pregnant women lacked a full understanding of malaria transmission and effective malaria prevention. They used traditional remedies and untreated bed nets, even while they respected the directives from healthcare providers to use modern prevention and treatment methods. Since that study, a systematic review of qualitative research on the uptake of malaria interventions among pregnant women in Africa highlighted the sociocultural factors that influence use of malaria prevention and treatment measures, including: locally defined disease categories, views of the vulnerability of pregnant women, the influence of healthcare workers (HCWs), and obstacles such as cost of services and distance to health facilities [23]. A small number of recent studies in African settings [24–28] have confirmed the importance of provider interactions with pregnant women, including messaging around use of preventative measures, and structural barriers, including travel to clinics and access to ITNs.

In the years since the Jharkhand qualitative study was published, relatively little additional research has been published on this topic in India. Two qualitative studies among pregnant women found a lack of concern about anaemia during pregnancy [29] and the importance of poor access to health care and personal emotions in choices regarding health care and health practices among low-income pregnant women [30]. At the same time, there is little evidence of government attention to improving knowledge and behaviours related to malaria prevention and treatment among pregnant women. In recent years, the focus of India’s National Vector Borne Disease Control Programme has been to test new options for treatment, implement new strategies to monitor for insecticide resistance, and scale up ITN use [31]. There has been training at a high level, but not among primary care providers [32]. Given signs of an increase in malaria cases in India, it remains of critical importance to better understand knowledge and practices among pregnant women and their care providers in India in order to improve efforts targeting malaria prevention and treatment in this population.

To contribute to these efforts, a comprehensive study on the knowledge and practice of malaria prevention and treatment among pregnant women and healthcare providers was carried out in two regions of India with a high malaria burden. The researchers aimed to build on the initial Jharkhand qualitative study with in-depth analysis of a large pool of qualitative data from pregnant women and different cadres of providers at a total of 7 sites in both Jharkhand and Chhattisgarh. The Jharkhand data included the initial qualitative data from pregnant women as well as additional data collected simultaneously from three cadres of HCWs; the Chhattisgarh qualitative data were collected at different sites which were used for a second study of the burden of malaria in pregnancy [33]. This is believed to be the most comprehensive study examining the views and behaviours of pregnant women in conjunction with healthcare providers’ perspectives ever conducted in India.

## Methods

### Study sites and population

Study respondents were recruited in 5 study districts in the states of Jharkhand (3 districts) and Chhattisgarh (2 districts) in East India, at a total of 7 collaborating hospital sites. In each of the 7 study sites, participants were recruited from two populations: pregnant women and HCWs. Pregnant women were sampled from those receiving antenatal care at, or living within the catchment area of, the collaborating hospital for the malaria in pregnancy burden of disease studies [13, 33]. Pregnant women were eligible for participation if they were ≥ 15 years of age and either currently or recently pregnant (within the last 2 years).

Health care providers encompassed 3 key groups: (1) healthcare providers at antenatal clinics (ANCs); (2) traditional birth attendants (TBAs); and, (3) auxiliary nurse midwives (ANMs). All 3 types of HCWs advise pregnant women in the months before delivery and are in a position to understand both their beliefs and behaviours; pregnant women typically make at least one visit to an ANC in their district, but also may seek care from a TBA and/or ANM. ANC-based providers have the most formal training and provide all routine ANC services, including malaria prevention and treatment. TBAs receive varying degrees of training, and remain widely used and respected in communities in Jharkhand and Chhattisgarh, largely due to persistent gaps in rural HCW availability and continued preferences for home-based deliveries in many communities [34–36]. ANMs, who generally provide primary care in community-level clinics, play an important role in maternal and child health and an increasing part in preventive health services at the community level [37]. All HCWs were recruited from among staff at the 7 study sites and their networks of TBAs and ANMs working at the community level.

Informed by previous experience conducting qualitative research, in which saturation is typically reached after interviewing 6–12 individuals with similar backgrounds, the researchers aimed to enroll at least 6 participants among pregnant women and ANC-based HCWs at each of the 7 clinic sites, along with as many TBAs/ANMs as possible (up to 6 in each group at each site). For pregnant women, this was accomplished by having members of the study team present at each of the study sites for 2–3 weeks and inviting all eligible pregnant women presenting at each clinic for an antenatal check-up to participate. All ANC-based providers at the hospital sites who met eligibility requirements were also invited to participate. Community-based ANC providers and TBA/ANMs who worked in the catchment area of a given collaborating facility were identified and contacted by that facility’s staff. Study staff then followed up with those who were interested in participating. All respondents provided written informed consent before data collection began.

Study sites were selected to represent both urban and rural areas. Sites in Jharkhand included an urban (Ranchi), a semi-urban (Gumla), and a rural (Konbir) location; there were 2 urban (Kondagaon, Rajnandgaon) and 2 rural (Dongargarh, Keshkal) sites in Chhattisgarh. All sites are characterized by a mixture of populations, including: (1) General Caste, comprised of traditionally higher castes and all persons not otherwise classified, and traditionally disadvantaged populations now given administrative recognition; (2) Scheduled Tribes, originally indigenous persons who have been largely integrated with mainstream society; (3) Scheduled Castes, primarily the historically lower castes; and, (4) Other Backward Castes, diverse groups historically viewed as lower castes. More detailed information on these districts and the 7 collaborating hospitals may be found in the published descriptions of the two primary malaria in pregnancy studies [13, 33].

### Data collection

In-depth interviews (IDIs) were employed to collect detailed, personal information from each respondent. Additional data collected via focus groups in Jharkhand have been reported elsewhere [22]. Local interviewers were trained on malaria knowledge, qualitative research methods and research ethics by members of the study team prior to data collection. Training included a detailed review of the interview guides to confirm clarity and understanding and role-playing to prepare the local team for situations that could arise during data collection.

Interviewers worked in teams of 2, with 1 person conducting the IDI and the other person taking notes. IDIs were conducted in Hindi using semi-structured question guides (1 for pregnant women, another for all HCWs, regardless of cadre) that covered specific topics and enabled open-ended exchanges and probing when unexpected responses emerged. Each IDI was audio-recorded and took 60–90 min to complete. Pregnant women were given a bed net for their participation.

Questions focused on health concerns and knowledge, views and behaviours regarding malaria prevention and treatment. Interviewers asked about traditional and modern approaches to malaria prevention and treatment, with follow-up questions on the availability and affordability of modern methods. IDIs with pregnant women included queries focused on individual behaviour, such as: (1) What do you do to protect yourself against malaria during pregnancy? and, (2) Have you been sick with malaria during this or any other pregnancy? If yes, what did you do? IDIs with HCWs covered the same topics, but with a focus on the knowledge and behaviours of pregnant women in the HCW’s community, with questions such as: What do pregnant women do to protect themselves against malaria during pregnancy? HCWs were also asked: At ANC visits, is malaria emphasized as a major concern for pregnant women?

The study was reviewed and approved by the Boston University Institutional Review Board, the Ethics Committee of the National Institute of Malaria Research in India, the Scientific Advisory Committee of the National Institute of Malaria Research, and the Health Ministry Screening Committee of the Indian Council of Medical Research. All respondents provided written informed consent prior to engaging in an IDI.

### Data analysis

The interviewer/note-taker team that conducted each IDI met daily during data collection with a bilingual study member to review the relevant recordings and confirm that Hindi language notes were accurate. These notes, which included notable quotations, were translated into English and verified by another bilingual study member through comparison with the IDI audio recordings. The coding and analysis utilized a similar thematic approach used for the initial qualitative study among pregnant women in Jharkhand, given the goal of building on those findings. A Boston-based co-author reviewed the themes and coding from the prior study and then coded the responses of the additional IDI data (from pregnant women in Chhattisgarh and HCWs in both states) using the same themes that had emerged from the earlier study. These were summarized in Microsoft (Redmond, WA, USA) Excel 2003 and a frequency analysis was conducted of themes as they related to key topics. A team of co-authors reviewed the analysis to ensure consistency across sites and participant groups. A response was included in the frequency analysis if mentioned by the respondent either spontaneously or in reply to follow-up questions. Responses of questionable interpretation were discussed with members of the Boston and India-based teams. Analysis included examination of differences by study population (pregnant women *vs* HCWs), state, site, caste, and level of education. Typical and atypical statements were identified for illustrative purposes.

## Results

### Characteristics of respondents

A total of 202 IDIs were conducted, 90 in Jharkhand in April–May 2007 and another 112 in Chhattisgarh in June–July 2008. These included 83 pregnant women, 32 and 51 in Jharkhand and Chhattisgarh (35.6 and 45.5% of all respondents), respectively, along with 58 and 61 HCWs, respectively: 24 (26.7%) ANC-based HCWs, 12 (13.3%) ANMs and 22 (24.4%) TBAs in Jharkhand and 31 (27.7) ANC-based HCWs, 14 (12.5%) ANMs and 16 (14.3%) TBAs in Chhattisgarh (Table 1). In each site, about 40% of respondents had less than a secondary education, while a similar proportion had secondary education; respondents were slightly more educated in Chhattisgarh than Jharkhand: in the former, 9% (vs 24%) had no education while 26% (vs 16%) had graduate level education. The majority of respondents were members of Scheduled Tribes or Other Backward Castes, though proportions differed by state, with Scheduled Tribes comprising 60% of respondents in Jharkhand, but less than half that proportion in Chhattisgarh. Monthly household income ranged from less than 1000 Indian Rupees (INR) ($25 at the time of data collection) to more than 5000 INR ($125), with about half reporting 4000 ($100) or less.

**Table 1:** Sociodemographic characteristics of study participants

Characteristic	Jharkhand n = 90 N (%)	Chhattisgarh n = 112 N (%)	Total N = 202 N (%)
Participant category			
Pregnant women	32 (35.6)	51 (45.5)	83 (41.1)
Auxiliary nurse midwife (ANM)	12 (13.3)	14 (12.5)	26 (12.9)
Traditional birth attendant (TBA)	22 (24.4)	16 (14.3)	38 (18.8)
Health care worker	24 (26.7)	31 (27.7)	55 (27.2)
Education			
None	22 (24.4)	9 (8.0)	31 (15.3)
Primary	10 (11.1)	21 (18.8)	31 (15.3)
Middle	5 (5.6)	13 (11.6)	18 (8.9)
Lower secondary	24 (26.7)	11 (9.8)	35 (17.3)
Senior secondary	15 (16.7)	25 (22.3)	40 (19.8)
Graduate	14 (15.6)	29 (25.9)	43 (21.3)
Caste			
General	12 (13.3)	24 (21.4)	36 (17.8)
Other backward castes	16 (17.8)	41 (36.6)	57 (28.2)
Scheduled caste	8 (8.9)	18 (16.1)	26 (12.9)
Scheduled tribes	54 (60.0)	28 (25.0)	82 (40.6)
Household monthly income, INR (SD)			
≤ 1000	6 (6.7)	10 (8.9)	16 (7.9)
1001–000	18 (20.0)	18 (16.1)	36 (17.8)
2001–3000	16 (17.8)	2 (1.8)	18 (8.9)
3001–4000	7 (7.8)	6 (5.4)	13 (6.4)
4001–5000	6 (6.7)	5 (4.5)	11 (5.4)
> 5000	37 (41.1)	50 (44.6)	87 (43.1)

The main results by theme are summarized below. Although stratified analyses showed no major patterns by caste or education, clear differences emerged by site and by population; these are highlighted where relevant. Selected statements are provided below, with additional statements on selected topics provided in Table 2.

**Table 2:** Illustrative statements of participants on malaria prevention and treatment among pregnant women

A. Attitudes	B. Knowledge	C. Behaviours
**i. Malaria in pregnancy as a top concern** *[Malaria is important] because the fever doesn’t cure quickly and it takes the lives of both mother and fetus.* –Pregnant woman, Jharkhand *If someone suffers from malaria, it may be difficult to save the life of both mother and fetus.* –Pregnant woman, Jharkhand *Malaria… [it] can cause the mother’s death and the baby can die.* –Pregnant woman, Chhattisgarh *The life of mother and baby may be in danger due to the unavailability of proper treatment in case of malaria [and] possibly abortion. Malaria is a fatal disease.* –ANM, Jharkhand *You can die. If [you] don’t get proper treatment, [malaria] can harm the child. If you don’t treat it, both mom and child can get malaria and both can die.* –TBA, Chhattisgarh **ii. Concerns about treatment for malaria in pregnancy** *[Medicines for malaria] may be dangerous. They may lead to stillbirth or the baby may suffer from weakness or jaundice.* –Pregnant woman, Jharkhand *Malaria medications will harm the baby, it will affect the child’s brain. Their brain will become weak and they will not be able to develop properly.* –Pregnant woman, Jharkhand *[A pregnant woman] should get advice from doctor. Yes, [malaria treatment] can be dangerous.* –Pregnant woman, Chhattisgarh *They [pregnant women] are scared to take it [treatment for malaria]. They think miscarriage can happen.* –ANM, Chhattisgarh *Those who know about it [treatment for malaria] think that it is harmful for fetus. Those who do not know anything about medications think that the fetus may be disabled, their organs will not develop, or there may be an abortion.* –ANC-based HCW, Jharkhand **iii. Trust in doctors regarding malaria treatment** *I will take medicine that is advised by the doctor and nurse. If there is a need to buy medicine, I will buy it….* –Pregnant woman, Jharkhand *Whatever the doctor will advise, I will take from the hospital, and purchase.* –Pregnant woman, Chhattisgarh *Pregnant women think malaria meds are harmful, especially Chloroquine, [but] if the doctor recommends it, she will take it, because they think that a doctor is god.* –TBA, Jharkhand *Yes….certain drugs are viewed more negatively than others…. [Pregnant women] think the baby will abort due to this. Yes, they will agree to take treatment on advice of the doctor because the doctor passes on the right information and they prescribe the right medicine. They [pregnant women] believe in the doctor.* –ANC-based HCW, Jharkhand *They [pregnant women] think they shouldn’t take it [malaria treatment], but if the doctor’s advice is to take it, then they will listen.* –TBA, Chhattisgarh *They ask [about] effects, but they think the doctor knows best, so they take it.* –ANM, Chhattisgarh **iv. Emphasis on malaria at ANC visits** *No [malaria is not emphasized]. They don’t tell us [about malaria], so I don’t understand.* –Pregnant woman, Jharkhand *No [malaria is not emphasized]. If they understood more about malaria they would definitely let us know.* –Pregnant woman, Jharkhand *They [care providers at ANC visits] just look at you and say everything is fine, and that’s it.* –Pregnant woman, Chhattisgarh *Special importance is not given to malaria. When pregnant women ask about it, then they will talk about it.* –ANC-based HCW, Jharkhand *Yes, importance is given to malaria because the fetus lives inside the mother’s womb.* ANC-based CHW, Jharkhand *No, doctors and nurses don’t say anything [about malaria].* –TBA, Chhattisgarh *Yes, because it can affect both the baby and child.* –ANM, Chhattisgarh	**i. Correct understanding about malaria transmission** *By mosquito bite, you get malaria.* –Pregnant woman, Chhattisgarh Malaria is caused by mosquito bite. –Pregnant woman, Jharkhand *[A] mosquito bite causes malaria. It causes fever which comes and goes.* –Pregnant woman, Jharkhand *[Malaria infection is] due to mosquito bite, due to female Anopheles mosquito bite.* –ANC-based HCW, Jharkhand *[Malaria infection is caused by] a female mosquito (Anopheles) bite.* –ANM, Chhattisgarh **ii. Misunderstanding about malaria transmission** *[Malaria is caused] by eating leftover food, eating decayed fruits, eating foods sold on a trolley….* –Pregnant woman, Jharkhand *[Malaria is caused] from dirtiness, from standing water in potholes because of dirty environment.* –Pregnant woman, Chhattisgarh *If a patient has a high fever and is sponged with a wet cloth, and if the same dried cloth is used on a healthy person, the healthy person gets malaria through the heat retained by the previous cloth.* –TBA, Jharkhand *[Malaria is caused from] drinking dirty water, allowing stagnant water near the house, by mosquito bite, [and] drinking polluted water.* –ANM, Jharkhand *[Malaria is caused from] using decayed/spoilt stuff, keeping unclean, drinking open water, holes filled with water.* –TBA, Chhattisgarh *If female Anopheles mosquito gives their larvae in clean water, and this water is used to drink, then they may suffer from malaria.* –HCW, Jharkhand **iii. Knowledge of methods to prevent malaria infection** *[To prevent malaria one] can use bednet, mortein coil and government sprays insecticide.* –Pregnant woman, Jharkhand *[How to prevent malaria?] Use mosquito coil, and bed nets.* –Pregnant woman, Chhattisgarh *[To prevent malaria], they should protect from mosquito; should use bednets; mortein coil should be burned.* –TBA, Jharkhand *[To prevent malaria, you need] mosquito net… and coils.* –ANM, Chhattisgarh **iv. Poor knowledge of malaria prevention** *Don’t know [how to prevent malaria].* –Pregnant woman, Jharkhand *[To prevent malaria], keep clean around the home, should wash hands with soap, take care with food, [and] drink boiled water.* –Pregnant woman, Chhattisgarh *Using dried or raw chiraita leaves (a medicinal plant) for making tea. It may be used anytime for 5* *days in a week as a drink which protects from malaria.* –*A*NM, Jharkhand *Make smoke by burning Karanj, Sinduwar leaves, [or] rice paddy dust, which repels the mosquitoes.* – TBA, Jharkhand *[You can] repel the mosquito by planting tulsi in the enclosed balcony and near window. Fresh air comes inside by planting fragrance flower, and then the mosquito can’t enter. Two drops of neem oil mixed [with] kerosene oil and used as a lamp for lighting. It repels the mosquito.* – TBA, Jharkhand *Eat carefully, keep things clean, don’t let water stand nearby….* –ANM, Chhattisgarh **v. Poor understanding of malaria treatment** *[Malaria] treatment may be serious. It can affect the fetus and may lead to fetal death.* –Pregnant woman, Jharkhand *Yes, it is dangerous for the baby … it will affect the baby in the womb…. The baby’s brain will become weak and will not be able to develop properly.* –Pregnant woman, Jharkhand *The born child may be weak in health, may be disabled or not normal…. It may put a curse on the baby.* –Pregnant woman, Chhattisgarh *Yes, they’re dangerous [malaria medications], especially chloroquine and Lariago tablets [a chloroquine formulation]. ….* –ANC-based HCW, Jharkhand *They do not take it [treatment] because it will affect the baby in a bad way. Yes, they [pregnant women] think so.* –ANM, Chhattisgarh *Yes, it [malaria treatment] can be harmful to the unborn child…. I haven’t seen, so I don’t know about it.* –TBA, Chhattisgarh	**i. Use of modern methods to prevent malaria** *[To prevent malaria] I use a bed net for sleep, and a coil before sleeping.* –Pregnant woman, Jharkhand *[For malaria prevention] I use a bed net…. So, if a mosquito still bites, what can I do?* –Pregnant woman, Chhattisgarh *Pregnant women use bed nets and coils. They use screens in the windows of the houses for protection from malaria.* –ANC-based HCW, Jharkhand *[Pregnant women] use bed nets during sleep; if they have no bed nets, they make smoke.* –TBA, Jharkhand *Educated people use bed nets and uneducated people should be given advice.* –ANC-based CHW, Chhattisgarh **ii. Poor availability of modern methods to prevent malaria** *Pregnant women do not use coils in this area because of their economic condition. They have a scarcity of money.* –Pregnant woman, Jharkhand *[Regarding bed nets]: They are not easily available as I live 8* *km away from the city.* –Pregnant woman, Jharkhand *I have heard about it [coil] and used it for 2* *days about 5* *months ago. I don’t use it now because I don’t have money.* –Pregnant woman, Jharkhand *[Regarding bed nets], you can get them easily at the store, [but] it is expensive.* –Pregnant woman, Chhattisgarh *I think it [insecticide spray] is expensive but…it is not even available here. If it were easily available here I would buy it, but the fact is it is not available here.* –Pregnant woman, Chhattisgarh *Bed nets? Poor people have a very hard time buying them.* –TBA, Jharkhand *[Coils for malaria prevention] are a little bit costly. Some people can’t afford them.* –ANM, Chhattisgarh **iii. Use of traditional preventive approaches** *Making smoke from paddy dust…..rub neem or karanch oil all over the body before sleeping…..* –Pregnant woman, Jharkhand *Smoke of neem leaves, cow dung. Drink pure water. Take care of food.* –Pregnant woman, Chhattisgarh *Boiling Tunj leaf and drinking its water for 3* *months will prevent malaria from occurring.* –ANM, Jharkhand *Bhui Neem leaf is ground with water and is given after filtering in the morning on an empty stomach for 3* *days for prevention. For prevention, if they drink this every month for 3* *days, then pregnant women will not get malaria. This is given to pregnant women for both prevention and treatment.* –TBA, Jharkhand *Two drops of neem oil are mixed with kerosene oil and used as a lamp for lighting; it repels the mosquitoes. Kunain herbal medicine is used as a drink in rural areas. They also drink “chiraita.”* –HCW, Jharkhand *Drink boiled water, use neem smoke… in the village they drink ayurvedic syrup,* *by putting neem leaves in boiling water and drinking it.* –ANM, Chhattisgarh **iv. Use of traditional remedies for malaria by pregnant women** *The Bhagat reads mantras and provides herbal medicine.* –Pregnant woman, Jharkhand *The Bhagat traditional healer does witch doctoring (Jhad Fouk) to cure malaria in return for rice and money.* –Pregnant woman, Jharkhand *Yes [there are traditional remedies]. You go to a traditional healer, get magical healing done, put on neem oil, and [use] neem leaves….* –Pregnant woman, Chhattisgarh *In this area, some do witch doctoring (Jhad*-*Fouk), some go to “Baidya”. Although they do not cure [malaria], but they still believe. They think that a pregnant woman is affected by some evil act like “Pang”.* –ANM, Jharkhand *The yolk of an egg is mixed with oil of “kunjari” and it is given as a drink. Kunjari is a fruit that grows in vine*-*like plants (climbing plants). Three drops of this is given as a drink … It may be given in the morning or evening on an empty stomach or full or partially full stomach*—*this cures malaria.* –TBA, Jharkhand *When pregnant women suffer from malaria then they are taken to the traditional healer. They cure malaria by lighting the earthen lamp, chanting mantras and doing witch doctoring. They also give herbal medicine [whereby] ground “Jhiti” root leaves with water and filtrate is given to the patient on an empty stomach.* –TBA, Jharkhand *[For malaria, you can] boil milk, mix with phurar stem, and drink it.* –TBA, Chhattisgarh *Put dhootvar plant’s on [the] head and wrap it. Brush teeth with neem brush.* –ANM, Chhattisgarh *[For malaria, you can] make drink of tulsi (mint) leaves, ginger, gud (sugar), black pepper.* –ANC-based provider, Chhattisgarh **v. Traditional remedy used with last fever** *I was afraid, because my mother*-*in*-*law told me that taking medicines during pregnancy may cause fetal loss. I drank turmeric in milk, because my mother*-*in*-*law [said it would cure me].* –Pregnant woman, Jharkhand *I had garlic and oil warmed and applied. … [I drank] boiled water, ate rice and roti [and then] took herbal medicines [which was my mother’s advice].* –Pregnant woman, Jharkhand *[I drank] boiled water, ate rice and roti…. No, I didn’t go [to the hospital].* –Pregnant woman, Chhattisgarh

### Attitudes towards malaria

Respondents’ attitudes regarding the importance of malaria as a health concern for pregnant women varied by state and population. Three-fourths of respondents in Jharkhand named malaria among the top three health issues facing pregnant women, including more than two-thirds of pregnant women. Most of these women, when queried about why they placed such importance on malaria, responded that it could cause harm or death to the mother and the baby. In Chhattisgarh, however, malaria was listed as a top health concern for pregnant women by only one in four respondents, including one-third of HCWs and just three pregnant women. When probed specifically about the importance of malaria, only one-fourth of pregnant women in Chhattisgarh acknowledged it as a concern. By contrast, the majority of HCWs in Chhattisgarh acknowledged that malaria was a serious health issue for pregnant women. (See Table 2, section Ai for direct statements by pregnant women and HCWs.) One exception was an ANC-based HCW in Dongargarh, who remarked: “it isn’t that important. I haven’t seen any pregnant women with malaria.”

Just over half of pregnant women (69% in Jharkhand, 46% in Chhattisgarh) indicated a willingness to try new methods of malaria prevention. While each modern prevention method elicited interest from at least some respondents, there was a particularly strong interest (nearly half of pregnant women) in trying ITNs among pregnant women in Jharkhand. Typical comments by these participants were, “With an ITN, mosquitoes will be repelled and I will enjoy sound sleep” and “I would like to try to use an ITN…because I now know that mosquitoes don’t even come near the net.” Pregnant women in Chhattisgarh were more likely to decline interest, as in this comment by a participant in Rajnandgaon, “I don’t need it right now and besides this, there is nothing else available.” Several claimed they would be interested if they could afford it. One participant in Kondagaon explained: “If we have money, then we use new methods.”

A majority of respondents expressed concerns about treatment for malaria during pregnancy, although HCWs described this concern more frequently (70% of respondents) than did pregnant women (42% of respondents). HCWs described a range of fears among pregnant women about malarial drug treatment, mainly potential harm to the baby. These included general poor outcomes (“the born child may be weak in health”), curses being placed on the baby, disability, and even abortion or miscarriage. About half of pregnant women had no opinion or said they did not know about treatment. Those that expressed worries or fear referred to similar dangers for the baby that were described by HCWs. Several mentioned possible fetal death. As one pregnant woman in Jharkhand (Konbir) explained: “…there may be miscarriage, and the baby will die, [and] then it causes emotional distress for the mother.” See Table 2, section Aii for further statements by participants.

Despite fears regarding treatment of malaria while pregnant, pregnant women nearly universally expressed confidence in whatever a doctor advised them to do. “I will take medicine that is advised by the doctor and nurse. If there is a need to buy medicine, I will buy it,” a pregnant woman in Jharkhand (Gumla) asserted. HCWs voiced the same view of pregnant women’s faith in doctors. A TBA in Jharkhand (Ranchi) noted that “pregnant women fear abortion by meds, but will take meds recommended by a doctor because they trust [the] doctor,” while another TBA in Chhattisgarh (Rajnandgaon) offered further insight: “After advice of a doctor, they take it. They don’t listen to the TBA [if the TBA says to take a malarial drug treatment] because we don’t know enough about medicines.” Table 2, section Aiii, contains more illustrative statements by pregnant women and HCWs on this topic.

Most HCWs (90% of those who responded) in both states indicated that malaria was stressed during ANC visits with pregnant women. Their responses included: “Yes, because there are two lives in one woman” (TBA in Chhattisgarh (Rajnandgaon)) and “They thought if malaria will happen then it creates a problem to them also (ANC-based HCW in Chhattisgarh (Dongargarh)).” One ANC-based HCW in Rajnandgaon offered a different perspective, noting that providers “look at the pocket of the person before giving advice.” Pregnant women generally voiced a different view: overall, 30% said malaria was emphasized, 12.5% in Jharkhand and 39% in Chhattisgarh. Most responses from pregnant women in Jharkhand were a simple “no” or “nobody gives us any information,” or “no one gives knowledge.” A number of pregnant woman from Chhattisgarh were more negative. As one explained: “They do not care about us. They do not give [malaria] any significance.” Another relayed that even though HCWs do “give advice…[but] we can’t understand the language they speak in.” Additional statements on ANC visits may be found in Table 2, section Aiv.

Three-quarters of pregnant women indicated that they had access to malaria information. Sources encompassed doctors, nurses, ANMs, radio, television, Integrated Child Development Services Scheme (ICDS) workers, community health volunteers (formally, these were Accredited Social Health Activists, known locally in Jharkhand as *Sahiya* and in Chhattisgarh as *mitanin*), community elders, and family members (usually women). When asked about the most reliable source of information about malaria, three-fourths of pregnant women identified doctors. This view reflected their overwhelming respect for doctors, and echoed their trust in doctors regarding malaria treatment, as described above (“whatever [the] doctor says will be true”). However, this opinion was expressed by only half of HCWs, who most typically named community health workers as most reliable for pregnant women. An ANC-based HCW in Chhattisgarh (Rajnandgaon) explained: “The *mitanin* can explain better than the doctor because they speak the language of the people [and] …spend more time with them so it will be more clear.”

### Knowledge about malaria transmission, prevention and treatment

A large majority of respondents correctly identified mosquito bites as the means of malaria transmission, including 88% of pregnant women and all but three (95%) HCWs. One in three, however, also described an incorrect transmission method, including a majority (63%) of pregnant women in Jharkhand (compared with one in four in Chhattisgarh). One-fourth of HCWs across both study sites also mentioned an incorrect method of transmission. Most incorrect methods suggested by respondents centred on a lack of cleanliness, with one pregnant woman in Kondagaon explaining that people get malaria “because of [a] dirty environment.” Food was commonly mentioned as a means of transmission, including food that was decayed, spoiled or touched by mosquitoes or flies. One pregnant woman in Ranchi stated that “if someone has malaria, pregnant women should not be near them because it spreads through touch and eating someone else’s food”. Water was also described as a potential source of transmission, through drinking polluted or “open” water and even through doing work in water. Some HCWs suggested that malaria was contagious among humans; an ANC-based HCW in Gumla described the practice of “keeping the malaria patient in a separate bed net…to stop the transmission chain.” Table 2, sections Bi and Bii, respectively, contain supplemental statements on correct and incorrect knowledge of malaria transmission.

Most respondents were also able to name a proven method of malaria prevention. When asked what could be done to prevent malaria in pregnancy, each pregnant woman in Jharkhand cited use of a bed net as a means of prevention and most (90%) noted at least one other method (usually mosquito coils), while 31% of pregnant women in Chhattisgarh did not name a proven prevention method. Other prevention methods beyond bed nets and coils were largely unknown to pregnant women: only 6 (4 from Jharkhand) initially mentioned insecticide spraying while none identified ITNs or IPT. Probing revealed that about one-third of pregnant women were aware of insecticide spraying as a prevention method, but few were familiar with ITNs (10%) or IPT (7%). All but three HCWs across the study sites mentioned at least one proven malaria prevention method. Bed nets were named by nearly all respondents and coils were identified by most, but other modern methods (ITN: 7%; insecticide spray: 19%; IPT: 7%) were mentioned infrequently. Further probing showed that awareness of these methods among HCWs was mixed, with about half indicating that they were aware of ITNs and an equal proportion aware of IPT (though only one-third of HCWs were aware of these methods in Jharkhand compared to two-thirds in Chhattisgarh). One ANC-based HCW in Chhattisgarh (Dongargarh) stated that pregnant women “shouldn’t use ITNs because [they] can affect [the] mother.” Further statements regarding these methods are provided in Table 2, section Biii.

Unproven prevention approaches were described by about half of all respondents. Respondents in Jharkhand noted them more frequently than those from Chhattisgarh (63 and 43%, respectively), while HCWs mentioned their use among pregnant women somewhat more frequently than did the pregnant women themselves (56 and 46%, respectively). Some of the methods mentioned may offer some protective benefit, such as burning items (neem leaves, bhusa wood, paddy rice dust) for smoke to drive off mosquitoes or use of Odomos mosquito repellent cream, although a range of unproven methods were also described, including using fans, boiling water, rubbing tree oils on the body, drinking teas, not eating cold foods, and cleaning with phenyl cleaners. One ANC-based HCW in Chhattisgarh (Rajnandgaon) described a general lack of malaria prevention: “Everywhere I’ve been there is no awareness about malaria. The women do all the housework and such [but] don’t use anything to protect themselves.” Table 2, section Biv, contains additional statements by participants.

Half of pregnant women in Jharkhand but only 19% of those in Chhattisgarh were aware that treatment for malaria existed, and just 3 were able to name a medication used to treat malaria. Nearly all (98%) HCWs knew of treatments for malaria, though some described treatments such as ayurvedic medicines, herbal syrups, and a traditional remedy of “yolk of egg…mixed with oil of “kujiri” and…given as a drink”. Statements illustrating understanding of traditional approaches to treat malaria are provided in Table 2, section Bv.

### Behaviours related to malaria prevention and treatment

Nearly all (99%) respondents in Jharkhand but only three-fourths in Chhattisgarh indicated that pregnant women in their areas used some modern method to prevent malaria during pregnancy. Bed nets were most frequently mentioned by these respondents (92%), although only 72% of pregnant women actually had a bed net in their house and used it regularly. Use of coils by pregnant women was indicated by three in four respondents in Jharkhand but only one-fourth in Chhattisgarh. Personal use of other modern methods was infrequently reported. Only three pregnant women said they used an ITN. One-third of respondents reported periodic spraying of insecticide by the government (indoor residual spraying (IRS), but not known in these words to most participants), although suggested that they did not feel it was done frequently enough. HCWs generally repeated the same information regarding malaria prevention among pregnant women. Regarding IRS, one ANC-based HCW in Chhattisgarh (Rajnandgaon) reflected that “it only gets sprayed [when] a politician or someone important comes to town”. Statements by HCWs on use of modern prevention methods by pregnant women generally and by pregnant women on their personal prevention strategies may be found in Table 2, section Ci.

There was general agreement among respondents that both bed nets and coils were available for purchase in their areas, but just under half of pregnant women indicated that bed nets (42%) and coils (40%) were not affordable. As one pregnant woman in Chhattisgarh (Dongargarh) stated when asked about a bed net, “we are poor people, how can we afford it?” Perceptions about the affordability of these two prevention methods varied between HCWs in Chhattisgarh and Jharkhand: 85 and 49%, respectively, said that bed nets were affordable, while 63 and 46%, respectively, felt that coils were affordable. An ANM in Konbir described these methods as “easily affordable for urban people, but villagers find [them] costly.” Table 2, section Cii, contains more statements by respondents on poor availability of and access to modern malaria prevention methods.

Half of all respondents described use of traditional methods for malaria prevention, but responses varied by site and population. Three-fourths of respondents in Jharkhand indicated use of traditional prevention approaches by pregnant women in their areas, compared to one-third in Chhattisgarh. While a majority (61%) of HCWs across both study sites described use of traditional prevention approaches by pregnant women, such use varied, as described by pregnant women themselves, across sites, with 66% in Jharkhand, and 17% in Chhattisgarh, respectively, indicating personal use of such a method. The most commonly mentioned practice was using smoke from burning something such as neem (*Azadirachta indica*) leaves, cow dung, or paddy rice dust to repel mosquitoes. A variety of other traditional methods was also described, including use of mustard (Brassicaceae) or karanj (*Millettia pinnata*) oil, drinking boiled neem leaf or ayurvedic syrup, washing food before eating, eating beng saag (a leafy vegetable), and planting tulsi (*Ocimum tenuiflorum*, a medicinal herb) near a window to repel mosquitoes. Neem leaf was said to be used in a number of methods, including this one described by a TBA in Konbir: “bhui neem [*Andrographis paniculata*] leaf is dried, ground into powder and given to [the pregnant woman] in [the] morning on an empty stomach for 3 days continuously.” Additional statements by participants on use of traditional malaria prevention methods are located in Table 2, section Ciii.

Most pregnant women revealed personal reliance on modern medicine to treat malaria during pregnancy, despite the fears they described and lack of knowledge about treatment (described above). About two-thirds of pregnant women respondents across the study sites reported having a fever or febrile episode during the current or a previous pregnancy. Of this group, most went to a hospital, doctor or clinic, although this was the case more often in Chhattisgarh (81%) than in Jharkhand (65%). All but one woman in Chhattisgarh and three-fourths of women in Jharkhand were given medicine during the episode; most did not recall the name of the medicine, but a mix of injections and pills was described. None mentioned being tested for malaria prior to treatment. When women who did not report a fever during pregnancy were asked what they would do if they got sick with fever or malaria, each respondent indicated that she would go to a doctor or hospital.

As with traditional prevention methods, use of traditional remedies for malaria by local pregnant women differed by site, with the majority (72%) of respondents in Jharkhand indicating their use in their communities compared to less than half (42%) in Chhattisgarh. Responses also varied by population: three-fourths of HCWs described use of traditional approaches by pregnant women in their communities, while only one-fourth of pregnant women indicated that they would use such remedies. Remedies described by respondents included drinking teas made from neem, tulsi and other plants, inhaling smoke from burning dried horse dung, and rituals performed by a traditional healer (see Table 2, section Civ for illustrative statements). Personal use of a traditional remedy while pregnant was described less frequently; only 7 women (about 15% of those who reported such an episode) described having used a traditional approach during a recent malaria or fever episode while pregnant. Statements by participants on use of traditional remedies for malaria are provided in Table 2, section Civ.

## Discussion

The results of this study provide broad insight into the attitudes, knowledge and behaviours related to malaria held by pregnant women in eastern India. Expanding the scope of a previous analysis to include both wider geographical coverage and the views of HCWs who work closely with pregnant women reinforces several findings from a previously published study on pregnant women in Jharkhand alone [22], while bringing to light a number of important differences and areas for potential action.

One consistent finding was limited knowledge about modern malaria prevention methods other than bed nets and coils. Not only were ITNs and IPT virtually unknown to pregnant women in both states, knowledge of these two methods recommended in international guidelines at the time of the study was also relatively low among HCWs. This highlights the need to improve education among HCWs and to utilize them better to spread awareness and knowledge of the options of effective malaria prevention methods for pregnant women, particularly those employed at ANC clinics or working in communities as ANC and TBA practitioners. Programmes should focus on educating HCWs about ITNs and ITP, and especially on how to describe their use to assure their communities about the effectiveness and safety of these approaches, and on potential opportunities for HCWs to distribute ITNs to pregnant women.

Issues with poor access due to cost of modern prevention methods that were widely known, bed nets and coils, were raised frequently by respondents across the broader study population. As has been found elsewhere in Asia and in Africa [23–25, 38], HCWs in the present study corroborated the views of pregnant women who described financial challenges in obtaining and consistently using these items. This issue was underscored for the research team when team members were unable to obtain ITNs in the data collection areas for distribution to pregnant women who participated in the study. Given indications that pregnant women were quite open to using ITNs if available, this poor access to prevention measures represents a lost opportunity to address the problem of malaria in pregnancy. Consideration might be given to distribution of ITNs or LLINs at ANC clinics, perhaps at each woman’s first visit during pregnancy.

These findings also affirm a near-universal respect for clinicians by pregnant women across these communities in eastern India, as also highlighted in other settings [25]. Even when women were unclear about a prevention or treatment approach, they expressed willingness to use whatever a clinician recommended, viewing this as more reliable than other sources of advice and guidance. This suggests that an opening exists to build on trust in clinicians to strengthen their role in helping pregnant women protect themselves, and yet one that must be accompanied by doing more to ensure that recommended and accepted malaria prevention methods are actually accessible to women.

This study highlights a number of differences between these two eastern states, perhaps most importantly that respondents in Chhattisgarh view malaria as less significant an issue for pregnant women than respondents in Jharkhand. While this may be related to the higher prevalence the two parent malaria studies observed in Jharkhand compared to Chhattisgarh, the difference was relatively small [13, 33]. National data suggest that both states have consistently been among India’s highest-burden states for many years [39]; together they contribute about 20% of all reported malaria cases nationwide [40]. Respondents in Chhattisgarh also showed generally poorer knowledge of malaria prevention and treatment methods than Jharkhand respondents. Interestingly, the use of traditional malaria prevention methods was reported more frequently in Jharkhand. It is clear that many factors determine continued use of traditional approaches (both prevention and treatment), including cultural preferences and perceptions of illness [41]. There is also evidence that some traditional approaches have proven biomedical effects. For example, there are many potential therapeutic uses of plants of the Neem tree [42]. Still, lower perceived significance placed on and more modest knowledge about malaria combined with the view that malaria prevention and treatment are not emphasized at ANC visits suggest that attitudes about malaria in Chhattisgarh may underestimate its risks to pregnant women and their children. This suggests a need to strengthen information regarding these risks in all areas of high risk, such as Chhattisgarh.

The study also identified differences in attitudes and perceptions between HCWs and pregnant women themselves. HCWs comparatively overstated both concerns with malaria treatment among pregnant women and the use of traditional treatments for malaria by that group. They also tended to view themselves and other providers as stressing the dangers of malaria with pregnant women, when the latter expressed the opposite opinion, that HCWs did not highlight such dangers or provide useful information regarding malaria prevention at ANC visits. It is recommended that efforts be made to strengthen the role HCWs play in emphasizing malaria prevention to their pregnant women patients, along with dispelling incorrect information about treatment, as misinformation is still present. Given the high level of trust communities place in clinicians, these findings suggest that HCWs are well poised to engage pregnant women with the aim of increasing use of both prevention measures and modern treatment methods where malaria is suspected. Another difference in views related to the most reliable source of malaria information for pregnant women, with the latter identifying doctors as the best source, whereas many HCWs suggested community-based health workers (TBAs, for instance), particularly in rural areas, because of their ability to better engage with pregnant women. This suggests that programmes to improve knowledge and practices related to malaria prevention and treatment may be most effective if they use messages that carry the authority and endorsement of clinicians but are delivered by HCWs based in communities.

Finally, the study results highlight that while malaria knowledge is generally high among HCWs, several important education gaps are evident that should be addressed. While nearly all HCWs named the correct mode of malaria transmission, many mentioned it among other incorrect causes. The same was observed for prevention methods, with unproven methods frequently being named alongside proven methods. Further education among HCWs about how malaria is transmitted and effective prevention methods, including use of ITNs and IPT, should help them deliver clear, consistent messages about malaria prevention to the women to whom they provide care. Given the importance of communication on these topics, as found in other settings [23], it is critical to ensure that HCWs are educated about effective malaria prevention for pregnant women. A number of HCWs also described a worrying practice of giving chloroquine to pregnant women suspected of malaria infection without having a confirmatory blood test, per national guidelines [17]. Pregnant women did not mention pre-treatment testing. Further investigation is warranted to determine if such practices are widespread at health facilities.

Several study limitations are important to note. First, it is important to acknowledge that the data are somewhat dated. However, since little has been published on this topic in India, the study involved collection of a large quantity of data from a variety of participants, and it remains critical to understand the knowledge and practices of pregnant women to inform new approaches to reduce the burden of malaria in pregnancy, we believe this study contributes valuable new insights. Second, although the study included respondents from varied settings (urban and rural) and populations (pregnant women and HCWs), the findings may not necessarily reflect views and behaviours elsewhere in India. There may also be views and/or behaviours that were not observed in the present study but that are relevant for improving malaria prevention and treatment. As respondents were recruited primarily among women attending or working at ANCs (with some residing in surrounding communities), it is quite possible that the views of women in comparatively marginalized, remote tribal populations may not be represented. Finally, it is possible that some questions may have been posed in potentially confusing ways, which may have led to misleading responses.

## Conclusion

These findings yield important understanding about how practices, knowledge, and attitudes related to malaria can vary, sometimes widely, across relatively close but separate locations and by stakeholder population. These results have implications for not just improving the effectiveness of implementing malaria prevention treatment programs in India, but for any geographically broad programme where modern protocols must compete with ingrained, traditional wisdom. Of particular concern are the general attitudes in Chhattisgarh about malaria as a health issue. While malaria prevalence among pregnant women in Chhattisgarh appears low, as it does in Jharkhand, it is still a serious illness with serious consequences for pregnant women and their children. Efforts should focus on strategies to help pregnant women understand that there is risk, even if it is relatively low, and to improve their local access to malaria prevention methods such as ITNs and LLINs in order to reduce the burden of malaria among this population.

## Abbreviations

WHO: World Health Organization
ITN: insecticide-treated bed nets
IPT: intermittent preventive treatment
LLINs: long-lasting insecticide-treated bed nets
HCW: healthcare worker
ANC: antenatal clinic
TBA: traditional birth attendant
ANM: auxiliary nurse midwife
IDI: in-depth interview
FGD: focus group discussion
INR: Indian Rupee

## References

[1] WHO. World malaria report 2017. Geneva: World Health Organization, 2017. http://apps.who.int/iris/bitstream/handle/10665/259492/9789241565523-eng.pdf;jsessionid=E1D66882B9A0C11C6EF57DA2B446BF62?sequence=1. Accessed 19 April 2018.

[2] WHO. Malaria SEARO. Delhi: World Health Organization, 2017. http://www.searo.who.int/india/topics/malaria/en/. Accessed 20 April 2018.

[3] WHO. Number of malaria cases. Geneva: World Health Organization, 2016. http://www.who.int/gho/malaria/epidemic/cases/en/. Accessed 20 April 2018.

[4] Wangdi K, Gatton ML, Kelly GC, Banwell C, Dev V, Clements ACA (2016). Malaria elimination in India and regional implications. Lancet Infect Dis.

[5] Sharma VP (2012). Continuing challenge of malaria in India. Curr Sci.

[6] Dash AP, Valecha N, Anvikar AR, Kumar A (2008). Malaria in India: challenges and opportunities. J Biosci.

[7] Gupta I, Chowdhury S (2014). Economic burden of malaria in India: the need for effective spending. WHO South East Asia J Public Health.

[8] Snow RW, Okiro EA, Gething PW, Atun R, Hay SI (2010). Equity and adequacy of international donor assistance for global malaria control: an analysis of populations at risk and external funding commitments. Lancet.

[9] Correa G, Das M, Kovelamudi R, Jaladi N, Pignon C, Vysyaraju K (2017). High burden of malaria and anemia among tribal pregnant women in a chronic conflict corridor in India. Confl Health.

[10] Guyatt HL, Snow RW (2001). Malaria in pregnancy as an indirect cause of infant mortality in sub-Saharan Africa. Trans R Soc Trop Med Hyg.

[11] Guyatt HL, Snow RW (2001). The epidemiology and burden of *Plasmodium falciparum*-related anemia among pregnant women in sub-Saharan Africa. Am J Trop Med Hyg.

[12] Guyatt HL, Snow RW (2004). Impact of malaria during pregnancy on low birth weight in sub-Saharan Africa. Clin Microbiol Rev.

[13] Hamer DH, Singh MP, Wylie BJ, Yeboah-Antwi K, Tuchman J, Desai M (2009). Burden of malaria in pregnancy in Jharkhand State, India. Malar J.

[14] WHO. Malaria in pregnant women. Geneva: World Health Organization, 2017. http://www.who.int/malaria/areas/high_risk_groups/pregnancy/en/. Accessed 4 Aug 2017.

[15] WHO. Guidelines for the treatment of malaria. 3rd Edn. Geneva: World Health Organization, 2015. http://www.who.int/malaria/publications/atoz/9789241549127/en/. Accessed 1 Oct 2017.

[16] Rogerson SJ, Desai M, Mayor A, Sicuri E, Taylor SM, van Eijk AM (2018). Burden, pathology, and costs of malaria in pregnancy: new developments for an old problem. Lancet Infect Dis.

[17] National Institute of Malaria Research, National Vector Borne Disease Control Programme, Government of India. Guidelines for diagnosis and treatment of malaria in India (2010). Delhi. 2010. http://www.nvbdcp.gov.in/Doc/Diagnosis-Treatment-Malaria-2013.pdf. Accessed 3 Oct 2017.

[18] National Institute of Malaria Research ND, National Vector Borne Disease Control Programme, Government of India. 2014 Guidelines for diagnosis and treatment of malaria in India. Delhi. 2014. http://www.nvbdcp.gov.in/Doc/Guidelines-Malaria-Diagnostic-Treatment-2014.pdf. Accessed 3 Oct 2017.

[19] Directorate of National Vector Borne Disease Control Programme, Ministry of Health and Family Welfare, Government of India. Diagnosis and treatment of malaria 2013. Delhi. 2013. http://www.nvbdcp.gov.in/Doc/Diagnosis-Treatment-Malaria-2013.pdf. Accessed 3 Oct 2017.

[20] Directorate of National Vector Borne Disease Control Programme, Directorate General of Health Services, Ministry of Health and Family Welfare and Government of India. Strategic Plan for Malaria Control in India 2012–2017: A 5-year strategic plan. 2012. http://nvbdcp.gov.in/Doc/Strategic-Action-Plan-Malaria-2012-17-Co.pdf. Accessed 3 Oct 2017.

[21] Directorate of National Vector Borne Disease Control Programme, Directorate General of Health Services, Ministry of Health and Family Welfare, Government of India. National Framework for Malaria Elimination in India (2016–2030). 2016. http://nvbdcp.gov.in/Doc/National-framework-for-malaria-elimination-in-India-2016%E2%80%932030.pdf. Accessed 3 Oct 2017.

[22] Sabin LL, Rizal A, Brooks MI, Singh MP, Tuchman J, Wylie BJ (2010). Attitudes, knowledge, and practices regarding malaria prevention and treatment among pregnant women in Eastern India. Am J Trop Med Hyg.

[23] Pell C, Straus L, Andrew EV, Menaca A, Pool R (2011). Social and cultural factors affecting uptake of interventions for malaria in pregnancy in Africa: a systematic review of the qualitative research. PLoS ONE.

[24] Hill J, Kayentao K, Achieng F, Diarra S, Dellicour S, Diawara SI (2015). Access and use of interventions to prevent and treat malaria among pregnant women in Kenya and Mali: a qualitative study. PLoS ONE.

[25] Rassi C, Graham K, King R, Ssekitooleko J, Mufubenga P, Gudoi SS (2016). Assessing demand-side barriers to uptake of intermittent preventive treatment for malaria in pregnancy: a qualitative study in two regions of Uganda. Malar J.

[26] Rassi C, Graham K, Mufubenga P, King R, Meier J, Gudoi SS (2016). Assessing supply-side barriers to uptake of intermittent preventive treatment for malaria in pregnancy: a qualitative study and document and record review in two regions of Uganda. Malar J.

[27] Pell C, Menaca A, Afrah NA, Manda-Taylor L, Chatio S, Were F (2013). Prevention and management of malaria during pregnancy: findings from a comparative qualitative study in Ghana, Kenya and Malawi. Malar J.

[28] Menaca A, Pell C, Manda-Taylor L, Chatio S, Afrah NA, Were F (2013). Local illness concepts and their relevance for the prevention and control of malaria during pregnancy in Ghana, Kenya and Malawi: findings from a comparative qualitative study. Malar J.

[29] Chatterjee N, Fernandes G (2014). ‘This is normal during pregnancy’: a qualitative study of anaemia-related perceptions and practices among pregnant women in Mumbai, India. Midwifery.

[30] Das A, Sarkar M (2014). Pregnancy-related health information-seeking behaviors among rural pregnant women in India: validating the Wilson model in the Indian context. Yale J Biol Med.

[31] Services DGoH, Welfare MoHF, Government of India. National Vector Borne Disease Control Programme: central website. Delhi. 2014. http://nvbdcp.gov.in/. Accessed 21 April 2018.

[32] National Institute of Malaria Research ND, National Vector Borne Disease Control Programme D, Government of India. National Vector Borne Disease Control Programme, District Level Trainers Training Programme, Udaipur: RNT Medical College. http://nvbdcp.gov.in/Doc/NIHFW-participants.pdf. Accessed 21 April 2018.

[33] Singh N, Singh MP, Wylie BJ, Hussain M, Kojo YA, Shekhar C (2012). Malaria prevalence among pregnant women in two districts with differing endemicity in Chhattisgarh, India. Malar J.

[34] Bajpai N, Dholakia R, Towle M. Increasing the availability of skilled birth attendance in rural India. Prepared for the National Rural Health Mission of the Ministry of Health and Family Welfare of the Government of India and UNICEF-India. New York. 2013. http://beta.global.columbia.edu/files/globalcommons2/IncreasingtheAvailabilityofSkilledBirthAttendanceinRuralIndiaWorkingPaper9.pdf. Accessed 24 April 2018.

[35] Bhattacharyya S, Srivastava A, Roy R, Avan BI (2016). Factors influencing women’s preference for health facility deliveries in Jharkhand state, India: a cross sectional analysis. BMC Pregnancy Childbirth.

[36] Dadhich JP (2009). The traditional birth attendants—Can we do without them?. Jour of Neonatology.

[37] Mavalankar DV, Vora KS. The Changing Role of Auxiliary Nurse Midwife (ANM) in India: implications for maternal and child health (MCH). Working Papers id:2755, eSocial Sciences. 2010. https://ideas.repec.org/p/ess/wpaper/id2755.html. Accessed 20 Oct 2017.

[38] Andrew EV, Pell C, Angwin A, Auwun A, Daniels J, Mueller I (2015). Knowledge, attitudes, and practices concerning malaria in pregnancy: results from a qualitative study in Madang, Papua New Guinea. PLoS ONE.

[39] Government of India, National Vector Borne Disease Control Programme, Directorate General of Health Services, Ministry of Health and Family Welfare. National Vector Borne Disease Control Programme: annual report of 2014–15.

[40] Kumar A, Valecha N, Jain T, Dash AP (2007). Burden of malaria in India: retrospective and prospective view. Am J Trop Med Hyg.

[41] Suswardany DL, Sibbritt DW, Supardi S, Chang S, Adams J (2015). A critical review of traditional medicine and traditional healer use for malaria and among people in malaria-endemic areas: contemporary research in low to middle-income Asia-Pacific countries. Malar J.

[42] Kumar VS, Navaratnam V (2013). Neem (*Azadirachta indica*): prehistory to contemporary medicinal uses to humankind. Asian Pac J Trop Biomed.

